# Awareness and attitudes towards anthrax and meat consumption practices among affected communities in Zambia: A mixed methods approach

**DOI:** 10.1371/journal.pntd.0005580

**Published:** 2017-05-12

**Authors:** Doreen Chilolo Sitali, Chisoni Mumba, Eystein Skjerve, Oliver Mweemba, Consolata Kabonesa, Mwinyi Omary Mwinyi, Luke Nyakarahuka, John Bwalya Muma

**Affiliations:** 1 Department of Disease Control, University of Zambia, Lusaka, Zambia; 2 Department of Health Promotion and Education, University of Zambia, Lusaka, Zambia; 3 Department of Food Safety and Infection Biology, Norwegian University of Life Sciences, Oslo, Norway; 4 Department of Gender Studies,Makerere University, Kampala, Uganda; 5 Department of Biosecurity, Ecosystems, and Veterinary Public Health, Makerere University, Kampala, Uganda; University of California San Diego School of Medicine, UNITED STATES

## Abstract

**Background:**

In Zambia, human anthrax cases often occur following cases of animal anthrax. Human behaviour has been implicated in this transmission. The objective of the study was to explore human behavioural patterns that may contribute to outbreaks of anthrax among affected communities.

**Methods:**

A mixed methods study was conducted in four districts of Zambia from November 2015 to February 2016. A cross sectional survey involving 1,127 respondents, six focus group discussions and seven key informant interviews with professional staff were conducted. Descriptive statistics on socio-demographic characteristics, awareness of anthrax, attitudes towards cattle vaccination and risk factors for anthrax and vaccination practices were run using STATA 12 for analysis.

**Results:**

Overall, 88% of respondents heard about anthrax, 85.1% were aware that anthrax is transmitted by eating infected meat and 64.2% knew that animals and humans can be infected with anthrax. However, qualitative data suggested that awareness of anthrax varied across communities. Qualitative findings also indicated that, in Western and Muchinga provinces, human anthrax was transmitted by eating infected beef and hippo *(Hippopotamus amphibious)* meat, respectively.

Although survey data indicated that 62.2% of respondents vaccinated their animals, qualitative interviews and annual vaccination reports indicated low vaccination rates, which were attributed to inadequate veterinary service provision and logistical challenges. While 82% of respondents indicated that they reported animal deaths to veterinary officers, only 13.5% of respondents buried infected carcasses. Majority (78.1%) of respondents either ate, sold or shared meat from dead animals with other community members. Poverty, lack of access to meat protein and economic reasons were cited as drivers for consuming infected meat.

**Conclusions:**

Health education campaigns must be intensified to reduce the risk of human exposure. Veterinary extension services should be strengthened and cold chain facilities decentralized in order to improve accessibility to anthrax vaccine. It is also important to involve the affected communities and collaborate with other disciplines in order to effectively tackle poverty, improve veterinary services and address inherent meat consumption practices within the communities.

## Author summary

Infectious diseases including anthrax are not only biologically determined but are also socially constructed and maintained. Yet, most of the interventions employed for the control of anthrax are largely derived from technical solutions with limited consideration of the social conditions existing in the affected communities. Interventions need to consider both technical and social factors in order to be appropriate and more acceptable to local communities. The authors conducted a survey to describe the socio-demographic characteristics, attitudes and practices of the affected communities. In order to gain an in-depth understanding of the socio-economic and cultural context, qualitative interviews were conducted. Quantitative results indicated that awareness of anthrax among respondents was high. However, attitudes towards eating anthrax infected meat and vaccination of cattle were poor. Qualitative interviews revealed that community members consumed infected meat due to poverty, economic reasons, cultural beliefs and perceived low risk of getting infected. These findings suggest that, in affected communities and possibly other similar settings, anthrax transmission is influenced by existing living conditions and inherent cultural practices. Therefore, the researchers suggest that health education messages should consider addressing these factors. More importantly, collaboration with other disciplines and involvement of local communities may improve effectiveness of control measures.

## Introduction

Anthrax is a neglected zoonotic disease of public health importance. The disease negatively impacts the livestock industry and causes worldwide concern because of its potential use as a biological weapon [1]. Anthrax does not only affect human health but also perpetuates poverty and causes emotional stress, especially among the poor populations whose livelihoods depend on pastoral farming [2]. This is because quarantine measures necessitated by disease outbreaks result in restricted livestock trade between areas, and hindrance of exchange of animals for drought power. Due to destruction of infected animals, household food security is affected and farmers experience financial losses [1].

Even if anthrax is distributed worldwide, it has been difficult to estimate its global incidence due to poor surveillance systems and unreliable reporting [3]. Globally, there is a general reduction in the number of reported anthrax outbreaks in livestock and human cases, although the disease still persists in most agricultural communities with tropical climates and poor socio-economic conditions [4]. The disease still exists in animals and humans in most countries of sub-Saharan Africa, Asia, in several European countries, America and certain areas of Australia [4,5,6].

In Zambia, anthrax predominantly occurs in traditional farming communities. The first case was reported in 1914 [1] and the first outbreak occurred in 1987 and claimed 4,200 hippos and 1,000 other animals. The number of human cases could not be ascertained due to diagnostic challenges. Siamudaala et al (2006) reported that between 1989–1995, 1,626 cases of suspected animal anthrax were reported and 51 were confirmed cases. An outbreak with 220 cases of human anthrax also occurred with a case fatality rate of 19.1% and another with 248 human cases with a case fatality rate of 7.7% in 1991.The outbreak in 2011 resulted in 233 suspected human cases with six deaths and approximately eighty hippos dying. The latest outbreak occurred between 17th September and 5th October, 2016 in Chikwa area of Chama district. About eighty human cases were recorded with twenty-five animals (including hippos and buffalos) dying [7]. Currently, anthrax is endemic in the western province [7] and occasional outbreaks of animal and human cases occur in Chama district of Muchinga province [7].

When the World Health Organization (WHO) established a Working Group on Anthrax Control and Research in 1990, Zambia was identified as a “model country” with the purpose of formulating improved anthrax surveillance and control programmes [8]. Since then, vaccinations, public awareness campaigns and quarantine measures have been used as essential intervention tools in anthrax control programmes in Zambia.

However, the problem of anthrax has persisted, especially in Western province where it has spread to other districts beyond Mongu and Senanga districts.

One of the drivers that may contribute to the persistence of anthrax in Zambia is human behaviour [9]. Because animals are an important asset to the communities affected, death of an animal may result in consumption of infected meat and use of animal products, potentially leading to infections. This is worsened by the fact that meat is a rare source of protein for most households, and a family may consume and sell some of the meat in order to salvage some losses from the death of the animal [1,9]. Because of this, anthrax is not only biologically determined but is also constructed and distributed as a result of community behaviours. Food safety behaviours ultimately influence exposure, vulnerability and consequences of anthrax disease [10].

Anthrax control measures must employ an understanding of the entrenched local knowledge, cultural factors and behaviours influencing transmission. A sensitive control strategy will provide effective communication that will help establish a bond of trust between responsible authorities and those who are potentially affected [11]. This may contribute to improved compliance to control measures and application of empirical evidence based on both technical and locally acceptable interventions.

Thus far, in Zambia, local knowledge and attitudes influencing transmission of anthrax are poorly explored and understood. Recommendations for anthrax control have largely depended on surveillance and management of biological agents, including medical treatment, contamination and decontamination [11]. Moreover, most published studies have focused on ecological, biomedical and epidemiological aspects rather than human behaviour. Furthermore, these studies have mostly used quantitative approaches which usually do not accord an in-depth understanding of contextual factors that influence behaviours. However, it has been acknowledged that cultural issues are always an important component of health, especially in agrarian communities [12]. Robert Ding Wall (2015) wrote in his commentary on the Ebola outbreak, “When technical interventions cross purposes with entrenched cultural practices, culture always wins.”

The objective of this study was to explore the human behaviours that may be associated with anthrax transmission in affected communities. The findings from this study will help programme implementers to design strategies that are culture specific and appropriate to community behaviours. An understanding of these behaviours will ultimately contribute to improved prevention and control measures which will enhance compliance to control measures by the affected communities.

## Methods

### Ethical considerations

The study received ethical approval from University of Zambia Biomedical Research Ethics Committee (UNZABREC) reference number 013-08-15. Permission to collect animal data was also obtained from the Provincial Veterinary Officers in the two provinces.

### Study sites

**Fig 1:**
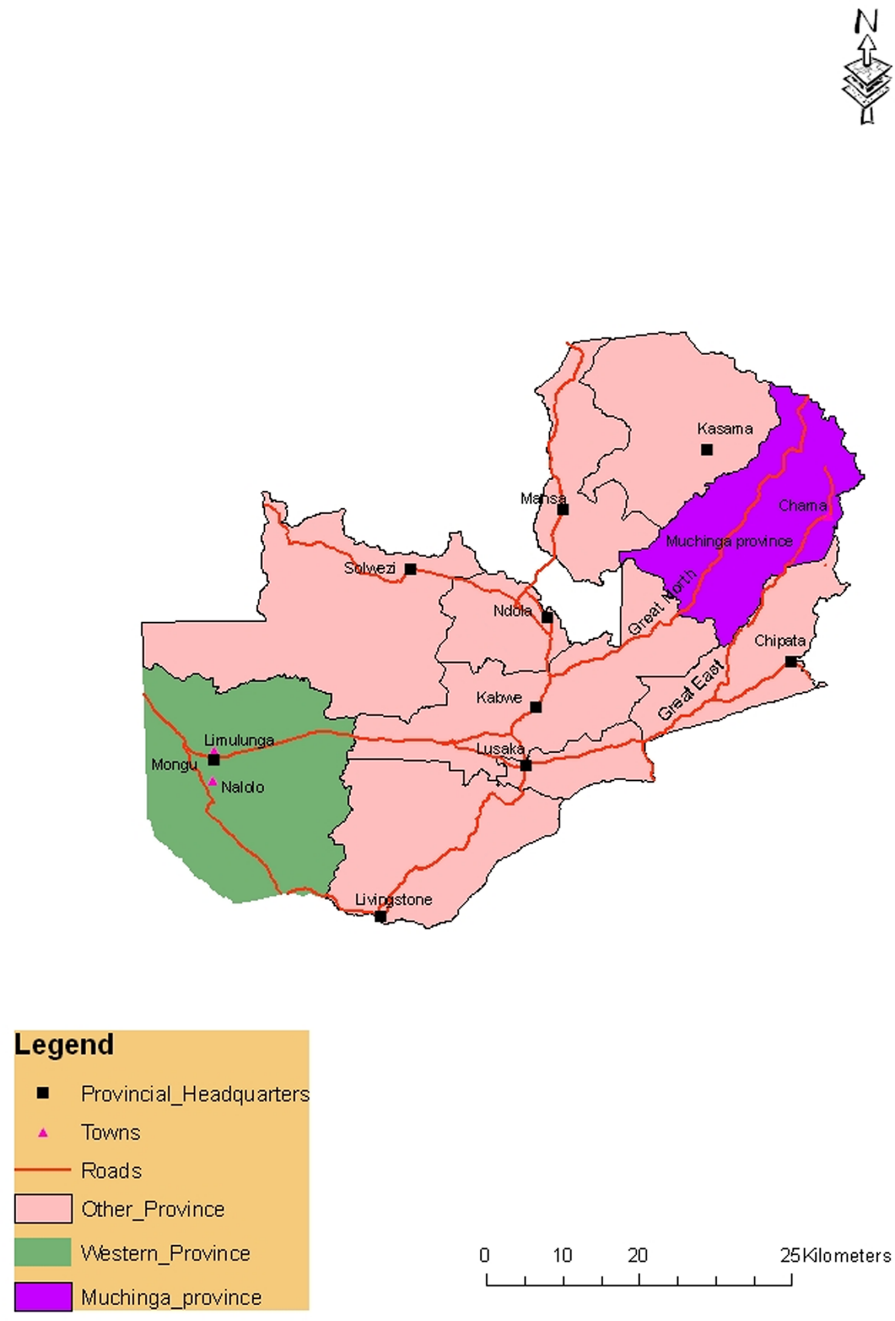
Map of Zambia showing study sites. Green color is Western province with Nalolo, Limulunga and Mongu districts. Purple color is Muchinga province with Chama district.

Four districts where outbreaks of anthrax usually occur in Zambia were selected, three from Western province (Mongu, Nalolo and Limulunga) and one from Muchinga province (Chama). Western province is dominated by the Barotse Floodplain of the Zambezi River. The plains are low-lying and flood during the rainy season. The floods usually leave behind deposits of organic materials along the riverbanks resulting in the ecological conditions described by Van Ness [6]. The province has a rural population of approximately 780,000 and an estimated cattle population of 751,000 [13]. Western province largely depends on livestock as a source of livelihood, food and draught power [1]. On the other hand, Chama is a remote district in the Muchinga Province of Zambia. It is served by one gravel road which comes from Lundazi. A dirt track connects the town to the South Luangwa National Park 200 km south-west, running parallel to the Luangwa River. Chama is an agrarian community dependent on subsistence farming (anonymous, 2010), and has a small cattle population of approximately 150,000 [13]. The largest part of the district is a game management area with an estimated total population of 104,000 [14].The study sites are shown on the map in Fig 1.

### Study design

A mixed methods study design was used; in which case a survey was concurrently conducted with focus group discussions (FGD), and key informant interviews (KII) between November 2015 and February 2016. This was done in order to get a deeper understanding of the underlying factors that influence attitudes and practices. The findings presented in this manuscript are only part of the main project that was undertaken.

### Data collection

#### Quantitative

A total of 1,127 household heads were interviewed using a structured questionnaire with mostly close-ended questions. All twenty-one (21) veterinary camps in the four districts were included in the study. A veterinary camp is the smallest administrative unit of a district. It is managed by a veterinary assistant and holds a maximum number of about five thousand herds of cattle. There were 836 respondents from Western province and 291 from Muchinga province. The questionnaire was translated into local languages, *Lozi* for Western Province and *Tumbuka* for Chama district. A pilot study was conducted in Mongu of Western province (not reported in this survey study). Before collecting data in each district, two to three veterinary camp officers were oriented to the questionnaire by the Principal Investigator in order to ensure consistency in data collection. Data was collected through face to face interviews. Questions asked involved whether respondents had ever heard of anthrax disease, knowledge of mode of transmission and who gets infected, their attitudes towards cattle vaccination, risk factors for anthrax and local veterinary services. Respondents were also asked about how they handled carcasses of moribund animals and whether any of their cattle or family members ever suffered or died from anthrax disease in the last twelve months.

#### Qualitative

In order to get an in-depth understanding of the context in which the disease occurs, qualitative data was also collected. Six (6) focus group discussions (FGDs)were held as follows: Chama (n = 1), Nalolo (n = 2), Mongu (n = 2) and Limulunga (n = 1).Mongu had two FGDs because it had twice the number of Veterinary Camps compared to other districts. Each focus group discussion (FGD) had an average of eight participants. Two (2) FGDs were held with female respondents only while the rest had mixed sex. Focus group participants were purposefully sampled from among survey respondents. Respondents who were willing to participate in the discussions were invited regardless of whether they owned cattle or not. FGDs were conducted using interview guides with open ended questions to allow respondents elaborate responses in their own words. Questions asked concerned what respondents knew about anthrax transmission, signs and symptoms of the disease in humans and animals, mode of transmission of anthrax, prevention measures and challenges that they face in controlling the disease, meat consumption practices, cattle rearing practices, cultural beliefs concerning anthrax disease and perceptions about local veterinary services. Respondents were also asked about how they handled carcasses and how they dealt with moribund animals. Discussions were held in local languages and were recorded on a digital recorder after getting verbal consent from the participants.

In addition key informant interviews with staff involved in anthrax control (n = 7) were conducted. Informants included veterinary officers (n = 3), health officers (n = 2), officers from the wildlife authority (n = 1) and from the agriculture sector (n = 1). Informants were asked what experiences they had with the local communities while implementing anthrax control programs, how they collaborated with other sectors, the challenges faced in controlling anthrax and how veterinary services were organized in their districts. Information from their experiences was cross checked with that of the FGD participants and quantitative data. Respondents’ confidentiality was observed by avoiding identifiers on the questionnaires and transcripts. Informed verbal consent was obtained from respondents before collecting information from them and before recording the discussions or interviews.

### Analytical methods

#### Quantitative

Descriptive statistics on socio-demographic characteristics, awareness of anthrax, attitudes towards cattle vaccination and risk factors for anthrax and vaccination practices were run using STATA 12 for analysis. Comparisons of socio-demographic characteristics, awareness, attitudes and vaccination practices between the two provinces were done using chi-square tests. Comparison of results was done between Western province and Muchinga provinces. This was because there were no significant differences noted between the three districts of Western province.

#### Qualitative

Audio files for focus group discussions were translated into English after which they were transcribed into computer files. After reading and re-reading the narratives, the data was transferred into Nvivo 12 for coding. Initially, broad coding was done using major themes that were derived from the topic guides. This was followed by further coding from the major themes into sub-themes. Framework matrices were then formulated by identifying key quotes under each sub-theme and summarising the narratives under each major theme. Framework matrices were used to cross check narratives from key informants with those of focus group participants in order to identify divergent or supporting views. Illustrative quotations that clearly represented the themes were used in the data results.

### Synthesis of quantitative and qualitative methods and data

Respondents for qualitative interviews were identified and sampled from among the survey respondents. Close-ended questions asked in the survey questionnaire were redesigned into open-ended questions in order to allow respondents to give meaning and add context to the responses. Qualitative interviews and discussions also allowed a deeper exploration of factors that could not be quantified through surveys and also provided an opportunity to gain an in-depth understanding of issues that could not be identified by the researchers. Quantitative and qualitative results were separately analysed but results were integrated during discussion. Apart from giving meaning, qualitative data was used to synergise, clarify or validate quantitative findings.

## Results

### Quantitative

#### Socio-demographic characteristics

The age range of respondents was 18–99 years, with a median age of 43.5 years. For educational level, the majority of respondents (85.9%) had never been to school while only 4.6% attained secondary education. Most of the respondents (89.3%) were self-employed. Significant differences in socio-demographic characteristics were noted between the two provinces as shown in Table 1.

**Table 1:** Comparison of socio-demographic characteristics of respondents between Western and Muchinga provinces.

**Characteristic**	**Category**	**Province**		**p value**
		Western n (%)	Muchinga n = (%)	
**Age (years) (n = 1,108)**	18–28	87 (10.6)	80 (27.9)	<0.01
	29–39	229 (27.9)	102 (35.5)	
	40–49	228 (27.9)	52 (18.1)	
	50–59	164 (20)	22 (7.7)	
	60–69	73 (8.9)	18 (6.3)	
	70–99	40 (4.9)	13 (4.5)	
**Sex (n = 1,112)**	Male	709 (85.7)	142 (49.8)	<0.01
	Female	118 (14.3)	143 (50.2)	
**Marital status (n = 1,113)**	Married	710 (85.9)	141 (49.3)	<0.01
	Single	117 (14.1)	145 (50.7)	
**Educational level** **(n = 1,119)**	None	720 (87)	241 (82.8)	0.001
	Primary	61 (7.4)	42 (14.4)	
	Secondary	45 (5.4)	7 (2.4)	
	Tertiary	2 (0.2)	1 (0.3)	
**Occupation** **(n = 1,123)**	Not employed	21 (2.5)	16 (5.5)	0.001
	Self employed	761 (91.4)	242 (83.4)	
	Public service worker	23 (2.8)	9 (3.1)	
	Informal employment	28 (3.4)	23 (7.9)	

As shown in Table 2, a majority (88%) of respondents had heard of anthrax across the two provinces. Most of respondents (64.2%) were also aware that both animals and human beings can be infected with anthrax. Although most of the respondents (83.5%) acknowledged that animal vaccination is an effective control measure for anthrax in Western province, the majority (63.3%) in Muchinga province did not know. A majority of respondents (86.4% in Western and 83.9% in Muchinga) knew that anthrax can be transmitted by eating infected meat.

**Table 2:** Comparison of awareness of anthrax among respondents in 2 provinces, given as numbers (%).

**Characteristic**	**Category**	**Province**		**p value**
		Western n (%)	Muchinga n (%)	
**Ever heard about anthrax (n = 1,127)**	Yes (n = 975)	738 (88.3)	237 (81.4)	0.003
	No (n = 152)	98 (11.7)	54 (18.6)	
**Awareness of who can be infected by anthrax (n = 1,125)**	Human (n = 228)	210 (25.0)	18 (6.2)	< 0.001
	Animal (n = 65)	14 (1.7)	51 (17.5)	
	Human & animal (n = 722)	543 (64.9)	179 (61.5)	
	Do not know (110)	67 (8.0)	43 (14.8)	
**Awareness of vaccination as an effective control measure (n = 1,127)**	Yes (n = 774)	685 (83.5)	89 (30.6)	< 0.001
	No (n = 353)	151 (18.0)	202 (69.4)	
**Awareness of mode of transmission (n = 1127)**	Eating infected meat (n = 966)	722 (86.4)	244 (83.9)	0.291
	Touching infected meat (n = 492)	383 (45.8)	109 (37.5)	0.01
	Slaughtering infected animals (n = 206)	135 (16.1)	71 (24.4)	0.002
	Handling infected animal products (n = 349)	275 (32.9)	74 (25.4)	0.018
	Touching infected animal feces (n = 210)	147 (17.6)	63 (21.6)	0.125
	Drinking infected milk (n = 290)	247 (29.5)	43 (14.8)	<0.01
	Don’t know (n = 99)	75 (9.0)	24(8.25)	0.707
**Source of information**	Radio (n = 260)	189 (22.8)	71 (25.2)	< 0.001
	Radio & Friends (n = 428)	378 (45.6)	50 (17.7)	
	Friends (n = 262)	150 (18.1)	112 (39.7)	
	Professional workers (n = 137)	94 (1.3)	43 (15.2)	
	Professionals & Radio (n = 23)	17 (2.0)	6 (2.1)	

However, there was no significant difference in awareness about eating infected meat as a common mode of transmission for anthrax between the two provinces. The most common source of information about anthrax in Western province was from radio and friends (45.6%) while in Muchinga province it was mostly from friends (39.7%).

Although majority of respondents were positive that animals can transmit diseases to humans, only 26.3% believed that eating infected beef was risky. These attitudes were significantly different between the two provinces, most notably the attitude towards local veterinary services. While most of respondents in Western province were positive about local veterinary services (89.7%), only 22.1% in Muchinga province showed a good attitude.

A majority (82.3%) of respondents in Western province reported that they vaccinate their cattle against anthrax. However, provincial vaccination records indicated that the majority of cattle farmers in Western province did not actually vaccinate their animals. Although most (34.5%) of the respondents across the provinces indicated that they reported cattle mortalities to the local veterinary authorities, only 13.5% burnt or buried the meat carcasses. Most (68.3%) of respondents participated in gutting of carcasses when an animal died in the community and (36%) shared the meat among their communities. Results are illustrated in Table 3.

**Table 3:** Community practices of relevance for anthrax in the two provinces, reported as numbers (%) in each province.

**Characteristic**	**Category**	**Province**		**p-value**
		Western n(%)	Muchinga n(%)	
**Vaccinates cattle against anthrax (n = 767)**	Yes (N = 689)	674 (82.3)	15 (5.2)	<0.001
	No (n = 78)	74 (9)	4 (1.7)	
**Treat dead animals from anthrax (1,209)**	Reported to vets (n = 924)	711 (85)	213 (73.2)	< 0.001
	Sold meat (n = 65)	55 (6.6)	10 (3.4)	0.048
	Ate meat (n = 184)	101 (12.1)	83 (28.5)	<0.001
	Used animal products (n = 20)	17 (2)	3 (1.0)	0.265
	Buried or burned(n = 16)	16 (2)	0 (0)	0.017
**Practices when animal dies from unknown disease**	Reported to local vet authorities (n = 389)	370 (44.3)	19 (6.5)	<0.01
	Sold meat (n = 261)	205 (24.5)	56 (19.2)	0.066
	Ate meat within family (n = 213)	200 (23.9)	13 (4.5)	<0.001
	Shared meat among community members (n = 406)	271 (32.4)	135 (46.4)	<0.001
	Buried /burned carcass (n = 152)	70 (8.4)	82 (28.2)	<0.001
**Activities participated in when animal dies in community (n = 1127)**	Gutting (n = 771)	636 (76.1)	135 (46.4)	<0.001
	Skinning (n = 18)	12 (1.4)	6 (2.1)	0.463
	Butchering (n = 168)	142 (17)	26 (8.9)	< 0.001
	Cooking (n = 242)	163 (19.5)	79 (27.1)	0.006

### Qualitative

Five major themes were generated from the focus group discussions and interviews namely;

➢ Awareness of anthrax
➢ Community attitudes towards anthrax
➢ Community practices
➢ Reasons for eating infected meat
➢ Challenges faced in controlling anthrax

#### Awareness of anthrax disease

The names by which anthrax disease is addressed suggest that respondents understood the common signs of disease and transmission route. In Western province, anthrax is called “*Lubete*”, which is the English translation for spleen. According to the respondents, the disease is called as such because one of the classical signs of anthrax in cattle is swelling of the spleen. In Muchinga province anthrax is called “*Chigwere*”, which is the English translation for hippo (*Hippopotamus amphibious*). The disease is called as such because the communities understand that it is transmitted by eating infected hippo meat. Participants were mostly able to identify signs of disease in animals.

*When the cattle die that is when we realize that it was suffering from anthrax*. *You will notice that the blood is coming out from the nose and at the back and at the tail* (referring to the anal orifice). *The disease spreads in the kraal fast*, *you see cattle just dying even 3 at once* (male participant, Mongu).

The majority of respondents were able to identify signs of cutaneous and gastro-intestinal anthrax.

*The disease of anthrax*, *what I have observed is that in humans there is a boil-sore which has no pus*, *even when you open it*, *it is just a swelling* (female participant, Limulunga).

None of the respondents identified or discussed inhalational anthrax. Generally, awareness of anthrax was poor among some communities, notably among female participants and villages where anthrax outbreaks had never occurred.

Participants mentioned that eating infected meat was the commonest route of transmission. One male participant described how he thought anthrax was transmitted from animals to humans, he said: *This disease of anthrax when our cattle died we did not bury*, *we ate*

Qualitative findings seemed to suggest two different origins of anthrax in the two provinces. A Wildlife officer working in Muchinga province explained that outbreaks of human anthrax commonly occurred following death of hippos in the Luangwa valley.

*What normally happens is when the hippos start dying*, *we have some fishermen and also in the human habitations people are mobile*. *So sometimes rather than our officers that go for patrols*, *these mortalities are discovered by nearby communities*. *So once they have discovered sometimes they by-pass the law*, *they would just start cutting the meat and start eating*. *Then the meat becomes too much for the communities living around those areas to consume so what they would do is to dry the meat and rush to where they could find the market* (Wildlife Officer, Chama).

For Western province, anthrax seems to originate from eating infected beef from moribund cattle as mentioned by a veterinary officer:

*… l mean human beings would get infected then we would follow and find that there are cattle that had died and people had eaten it and eeh got infected*. *That is the situation in Western Province*. (Veterinary Officer, Mongu)

#### Attitudes of community towards anthrax disease

Professional workers involved in anthrax control indicated that most community members had negative attitudes towards not eating meat from moribund animals. Professional workers narrated that one of the challenges they faced in anthrax control was failure to convince community members against eating infected meat as indicated below.

*I think according to my own view*, *l think one of the things that makes them not to comply*, *it’s just the mind-set*. *Yes*, *the mind-set*, *so for us to convince them that meat can actually kill*, *we need to work very hard*, *yes*. (Veterinary Officer, Limulunga)

In addition, interviews showed that most cattle farmers had a negative attitude towards cattle vaccination against anthrax. One veterinary officer narrated that in his veterinary camp, farmers would usually avoid taking their animals for vaccinations for fear that the disease was going to be introduced into the animals and cause them to die.

*They hindered the progress of vaccinations*, *others would even hide their animals they didn’t bring it to the crash pane for fearing that they might die after vaccination*. *…so they think by putting a vaccine in an animal would make it die*, *get an infection of that disease and die*, *yes*. *Those are the hindering points we are facing*, *for fear of the animal dying after vaccination…*.(Veterinary Officer, Nalolo)

The above quotation seems to be supported by what focus group participants felt about animal vaccinations. One male participant in Western province said:

the veterinary have vaccinated them though there has been no change observed …. that may be the vaccines do not work on the disease.

Veterinary staff indicated that traditional beliefs and practices seemed to have an influence on community attitudes as indicated below.

*l mean*, *this is meat that we have been eating for years and years*, *our ancestors have been eating and now you say it’s infected*, *it’s making us sick*, *me l refuse because as far as we are concerned*, *there are no graves for cattle*. (Veterinary Officer, Mongu)

Such beliefs and attitudes clearly influenced how people received and interpreted health education messages that they received from professional workers.

#### Community practices

Apart from cultural beliefs, there are traditional practices that have been implicated in anthrax transmission. One such practice is the ‘*Mafisa*’ traditional practice. In this practice, cattle owners give their cattle to other people to herd in order to spread the risk of dying. Such animals are used for manuring, and draught power. Using the same practice, cattle herders are paid an agreed number of cattle as payment. This practice in turn empowers those families without cattle to eventually own some. However, in the event that cattle dies while under the care of the herder, there is need to produce evidence. Therefore, meat and the animal skin are dried and taken to the owner as evidence for the dead cattle. So if cattle died from anthrax, it meant that along with the meat, the disease was transferred to a new area. One respondent explained what happens when cattle on ‘*Mafisa’* dies:

*If the owner of cattle is far away*, *the meat is cut into threads and dried*. *The skin is also dried so that the owner can find it*. *The skin is stored because it is evidence for the dead cattle*. (Female respondent, Mongu)

Another practice is associated with the Lozi traditional ceremony called ‘*kuomboka ceremony’*. The ceremony takes place at the end of the rainy season when the upper Zambezi river floods the plains of the Western province. Due to flooding, the ‘*Litunga’*, the king of the Lozi’s and his subjects move upland along with their livestock and belongings. They stay upland until the waters have subsided. Therefore, cattle stays upland for manuring the fields. This movement of cattle between the areas facilitates the spread of anthrax. In case, cattle dies while on the upland, meat has to be dried and taken to the owner in the plains as evidence, therefore spreading the disease. This was illustrated by one veterinary officer who said:

… the owners of the cattle stay in the plain, so when the animals go in the upland, they stay with other people there for manuring the fields, so you find that an animal got affected in the upland, the meat has to be transported to the plain. So you have an outbreak from different place from where it died.

#### Meat consumption practices

Respondents discussed a number of meat consumption practices that they thought could be implicated in the transmission of anthrax in the community. One such practice was where community members who helped in skinning and gutting dead cattle each received a share of meat. The practice was called ‘*Maleu*’. This practice exposes more people to the disease other than the owner of the cattle. A female respondent explained what happened when an animal died in her community:

Yes, what we do is that if the veterinary officers do not come at that time we skin after skinning just there where the cattle died from, we start to share among ourselves, there is meat that they give the skinners.

Sometimes meat was shared among community members, sold, or exchanged with other items

Dead cattle, we help ourselves by eating it, some meat we sell and keep the money so that may be able to buy another animal. Sometimes we burn it.

Other practices discussed included boiling the meat for the whole day to render it safe for eating, or cutting out and throwing away parts of the meat which were believed to be infected by anthrax and eating the rest of it.

*Us* (village) *we are of the belief that*, *meat when it is cooked then the disease is killed*, *it cannot be transmitted* (female FGD participant)

#### Reasons for consuming infected meat

Reasons cited for consuming meat included, lack of education, cultural beliefs, poverty and economic reasons. Poverty and lack of health education were cited as the most common causes of eating infected meat in all the six FGDs. A female respondent elaborated how hunger and lack of health education affected their meat consumption behaviour.

Those who eat meat is due to misery caused by hunger, there is draught in Lozi land, rains do not fall, crops are not doing well, thus there is no food, so due to lack of enlightenment and hunger, they eat dead cattle’s meat.

Other than lacking awareness and hunger, geographical location also seem to influence consumption behaviours of these communities. The remote location of the communities make it difficult for them to access certain facilities such as butcheries where they can buy safe meat. Chama district is one such area.

…. We do not have butcheries here, we suffer because we do not have butchery facilities here, that is why whenever we hear of information on meat, even for small animals you go and get it to eat.

Chama district is a game management area (GMA) and therefore rearing of domestic animals is a challenge because of wild carnivores. The communities therefore have problems accessing meat protein.

Both professional workers and community respondents pointed out that cattle was the main source of wealth and livelihood [15]. Respondents showed that most people consume or sell meat from infected carcasses due to economic reasons. Therefore, when cattle die, most families are forced to salvage some losses by eating or selling the meat. This was explained by a female participant during discussions on the impact of anthrax on the livelihoods of people:

It does not feel good because that is where you had put all your hopes and thoughts altogether. Now that it dies, that is why you see that many people fail to bury so that they can sell some meat as something to get comfort from. That is what causes that they sell meat from cattle which died on its own.

Dependence on cattle for livelihood means that families have to put up some cost recovery measures whenever they lose cattle

A number of expressions indicating the economic impact of loss of cattle were made by respondents in most focus group discussions in Western province. One male participant said:

*Cattle here in Lozi land is our mine because we do not work*, *it is our copper* (copper mining is the major source of the country’s economy).

Another female respondent said:

It feels bad because it is an asset that helps us, when you sell it, it helps you but when it dies, it feels bad, it is the same as losing a child.

These study findings indicate the economic importance of cattle, especially in Western province and the emotional stress that cattle losses cause in the communities.

Apart from poverty and lack of education, it was found that entrenched cultural beliefs and practices were contributing to resistance in behaviour change. In describing challenges that they face in anthrax control, most key informants indicated that most communities had certain beliefs and practices that contributed to the transmission of anthrax disease. Below is an example of some of the responses that veterinary officers in Western province commonly received from community members:

l mean, this is meat that we have been eating for years and years, our ancestors have been eating and now you say it’s infected, it’s making us sick, me l refuse because as far as we are concerned, there are no graves for cattle.

Such beliefs and attitudes clearly influence how people receive and interpret health education messages that they may receive from professional workers.

This is clearly demonstrated by what by one participant said during a focus group discussion.

Us Lozis we don’t slaughter cattle for relish unless it dies on its own. Dead cattle, we help ourselves by eating it, some we sell and keep the money so that may be able to buy another animal, sometimes we burn it.

It is shown here that meat consumption patterns are equally shaped by cultural beliefs. In Western province, cattle are mainly kept for draught power, manure and for wealth and prestige.

Other reasons for consuming meat is that people do not perceive eating meat as risky because they did not observe obvious signs of disease in those who ate the meat. One participant said:

…what causes that we eat …there is nothing that happens on our bodies when we eat all of the meat …

Animal vaccination is an effective preventive measure for anthrax. Animals should receive vaccinations every year in order to avoid infections. However, animal vaccination levels have been low in Western province due to various reasons. One of the reasons described by one informant in Western province was lack of knowledge and poor attitude by cattle farmers. However, a senior veterinary officer pointed out that most farmers do not understand what diseases their animals are vaccinated against. In Zambia, it is the responsibility of the farmer to buy their own vaccine and have their animals vaccinated against anthrax. However, other vaccinations like Foot and Mouth Disease, Black leg and Contagious Bovine Pleural Pneumonia (CBPP) are given freely. But because most farmers are of low education or are not well informed, they think that the free vaccinations also cover anthrax disease. The other reasons for the low vaccination were failure to afford the cost of the vaccine and poor access to the vaccine and logistics. A senior veterinary officer explained a practical scenario in Western province and said:

*It is up to the farmer to vaccinate his animals but to start with even if they know*, *they cannot afford*. *Sometimes they may want to protect their animals but now the vaccine might not… is not readily available*, *what is involved to get your animals vaccinated it’s not easier*. (Veterinary Officer, Mongu)

Similar concerns were raised by all key informants from the veterinary sector that they faced challenges of poor funding, inadequate transport, and lack of cold chain facilities in remote areas, poor staffing and lack of accommodation and office space for them to work effectively. Because of this, they are not able to vaccinate cattle as recommended. They mentioned that government does not fund anthrax vaccinations unless there is an outbreak. Therefore, free vaccinations are only conducted when there are outbreaks. This scenario seemed to create frustration among cattle farmers and has contributed to certain misconceptions. One misconception is that the vaccine makes cattle to get infected with anthrax and die; therefore farmers avoid taking their animals for vaccination.

There were several challenges faced by both control officers and the community in getting animals vaccinated. Most community participants also mentioned poor extension services, lack of funds to pay for vaccination and lack of government support. They also kept their animals from being vaccinated for fear of their animals dying because veterinary officers mostly conducted vaccinations when there was an outbreak. They also indicated that most of their communities had no resident veterinary officers within their localities due to lack of accommodation.

## Discussion

Human behaviour has a significant role in influencing anthrax transmission. This behaviour is influenced by the knowledge, attitudes and practices of affected communities. This study aimed at exploring awareness, attitudes and meat consumption practices of communities affected by anthrax in Zambia.

Although the Zambia Demographic and Health Survey (2014) shows that the proportion of males and females are almost equal, there were significantly more male than female respondents in the study. This can be attributed to the fact that females are less likely to be listed as household heads but also that males dominate the role of herding cattle.

The study also found that most respondents (85.9%) have not received any form of education. Education influences one’s access to information and ability to comprehend health messages. This means that the majority of both male and female respondents are more likely to have poor access to media information and poor comprehension and compliance to health education messages. The study showed that most respondents accessed information from friends rather than public media. The Demographic and Health Survey (2014) shows that 60.1% and 41.3% of people in Western and Muchinga provinces do not have access to any form of media such as radio, TV or newspaper. This situation is likely to interfere with public health messages as community members share misconceptions and myths surrounding the disease. This is as observed by Taverne [16] who postulated that disease epidemics arrive ‘ahead of themselves since interpretations and the social effects usually precede the disease itself.’

The survey results indicated good awareness about anthrax among respondents. Both male and female respondents also indicated an understanding of the susceptibility of animals and humans to anthrax, the signs and symptoms of anthrax in dead animals and sick human beings, seasonal outbreak periods, common transmission routes and the importance of vaccination as a preventive measure. However, focus group discussions revealed that some communities had poor understanding of the disease. These study findings are consistent with those of Gombe *et al* [6] and Mebratu *et al* [4]. On the contrary, Opare‘s study [9] showed that most respondents did not know the causes of anthrax but recognised the signs and symptoms of anthrax and the potential effectiveness of vaccinations.

Even if questionnaire data showed that 89.8% of respondents vaccinated their animals, qualitative information suggested that very few farmers had their animals vaccinated. This was confirmed by provincial vaccination records that indicated a vaccination rate of less than 10%. Both focus group participants and key informants confirmed that there were challenges with animal vaccinations. Veterinary staff pointed out lack of funding, transport and logistical challenges and myths about vaccinations within the communities as major factors. On the other hand, FGD participants highlighted inability to pay for vaccination, lack of cold chain facilities and poor veterinary services in their communities. A similar situation seems to obtain in Ghana where Opare [9] indicated that high levels of knowledge about vaccination had not been actualised into practices by farmers in Tamale Municipality. Equally, a review of challenges faced by sub-Saharan countries in controlling CBPP disease in Western province identified failure of veterinary services as one major challenge expressed by most staff who were involved in the control [17]. Likewise, in a review paper by Siamudaala et al (2006), inadequate technical and administrative support, erratic funding and supply of logistics were cited as major constraints of anthrax control in Zambia [1].

Similarly, high levels of knowledge were not found to be consistent with the attitudes and perceptions of respondents in this study; while quantitative data indicated that most respondents reported animal deaths to veterinary authorities, focus group discussions and key informant interviews revealed that the majority of community members believe that meat from cattle that die of ‘natural’ causes can be consumed because that has been the practice in their communities. They also believe that when meat is cooked for a long time, the bacteria die and the meat is safe to eat. Contrary to this finding, the study done by Gombe *et al* in Zimbabwe revealed that respondents disagreed with statements that overcooking infected meat kills anthrax bacteria [6]. Respondents also indicated that they did not think eating meat from moribund animals was risky because they usually do not see any symptoms among themselves whenever they consume meat from diseased carcasses. This perceived low susceptibility to anthrax is likely to lead to risky meat consumption behaviours. This perception seems to be consistent with the propositions of the Health Belief Model. The Health Belief Model proposes that persons who perceive a low risk of developing a health problem are unlikely to engage in behaviours to reduce their risk of developing the particular health problem [18].

The study also showed that when cattle died due to unknown disease or anthrax, respondents informed veterinary officers who, on most occasion’s failed to respond due to logistical challenges. Therefore, most respondents would eat, sell or share meat (36%) among the community members. There was also a high proportion (68.3%) of respondents who participated in gutting animals and cooking (25%) meat. According to qualitative data obtained, these practices were perpetuated by poverty and lack of access to meat protein. Furthermore, both key informants and FGD participants indicated that cattle were the main source of livelihood for the people of Western province. Therefore loss of cattle leads to economic losses and increases the likelihood of selling infected meat in order to make financial recoveries. This is worsened by the fact that farmers are not compensated for cattle losses. Similar practices and reasons have been cited in other studies by Mebratu et al [4], Gombe, *et al* [6], Munang’andu *et al* [18], Opare *et al* [9]. These findings are consistent with the arguments of the theory of political economy advanced by Sydenstricker, Goldberg and Davey- Smith who argue that diseases are not just a part of biology but are socially produced and distributed [19]. According to these theorists, the socio-economic conditions and political context of a particular society create differential opportunities that create social inequalities and therefore place people in different advantages and disadvantages.

The study findings show that awareness varied among anthrax affected communities. The study also shows that vaccination rates in these communities are generally low due to a number of challenges such as cultural beliefs and perceptions that influenced how respondents treated dead carcasses. The study clearly indicated that anthrax was transmitted by eating infected cattle meat in Western province while in Muchinga province anthrax was transmitted through eating infected game meat. Lastly high risk practices such as selling, eating, or sharing infected meat were found to be common among community members. These risk factors are influenced by socio-economic conditions of the communities and poor veterinary services.

In view of the above findings, it is important for the Ministry of Livestock and Fisheries to strengthen health education campaigns in the affected communities. These campaigns should take a One Health approach, with the involvement of the community, social scientists and health personnel. Social scientists will enable an in-depth understanding of contextual factors and formulation of culturally and context specific interventions that are likely to be more acceptable by local communities. Community involvement will enhance ownership and acceptability of control interventions. Furthermore, there is need for the veterinary department to strengthen extension services in the communities. This will enhance access of veterinary services for the community. Using the One Health approach will also enable maximum use of resources, for example, challenges of the cold chain facilities can be overcome if the veterinary department and health work together. Lastly, government needs to prioritise anthrax control and revisit some of its policies on control. For example, vaccine stocking should be decentralised to make it more accessible to the communities and funding for logistical support should be provided in order to make animal vaccinations more accessible.

### Conclusion

The origin of human anthrax in Western province is infected cattle while in Muchinga it is infected game. The study found that awareness of anthrax varied across communities with some communities being more aware of the disease than others. It was also found that most respondents do not vaccinate their animals against anthrax. Meat consumption practices were found to be high risk for anthrax as most respondents either consume, sell, or share infected meat after reporting death of cattle to veterinary authorities. These practices are mostly driven by social factors such as poverty and socio-economic losses incurred by loss of cattle and cultural practices. It is therefore important that a holistic approach to control the disease is taken by using a One Health approach. This will require political commitment and government support.

### Limitations

The study findings may not be very applicable to areas with different social conditions due to lack of random sampling. The findings may not be have systematic errors due to self-reporting and recall bias. However, the study endeavoured to ensure internal validity and reliability by triangulation of quantitative and qualitative findings. Instead of contradicting, the two components seem to be clarifying aspects of data that do not seem to be in agreement. Lastly, the study was not designed to estimate the prevalence of anthrax in the affected communities in order to confirm the magnitude of the problem in humans. Therefore further research to ascertain the extent of the problem in humans should be undertaken.

## Supporting information

S1 Checklist STROBE checklist. (DOC)
